# Hepatoprotection of Lycii Fructus Polysaccharide against Oxidative Stress in Hepatocytes and Larval Zebrafish

**DOI:** 10.1155/2021/3923625

**Published:** 2021-02-18

**Authors:** Fang Zhang, Xia Zhang, Yutian Gu, Min Wang, Sheng Guo, Jiazheng Liu, Xiaofei Zhang, Zihan Zhao, Bowen Qian, Yichao Yan, Li Yu, Chunlei Xu, Chunmei Liu, Funing Cao, Dawei Qian, Jin-ao Duan

**Affiliations:** ^1^Jiangsu Collaborative Innovation Center of Chinese Medicinal Resources Industrialization, School of Pharmacy, Nanjing University of Chinese Medicine, Nanjing 210023, China; ^2^School of Pharmacy, Ningxia Medical University, Yinchuan 750004, China; ^3^Key Laboratory of Hui Ethnic Medicine Modernization of Ministry of Education, Yinchuan 750004, China; ^4^Hong Kong Polytechnic University, Hong Kong 999077, China; ^5^School of Medicine, Nanjing University of Chinese Medicine, Nanjing 210023, China; ^6^State Key Laboratory of Quality Research in Chinese Medicine, Macau Institute for Applied Research in Medicine and Health, Macau University of Science and Technology, Macau 999078, China; ^7^Jiangsu Golden Thinking Software Co., Ltd., Nanjing 210042, China

## Abstract

Scavenging of oxidative stress by antioxidants may provide a therapeutic strategy for nonalcoholic fatty liver disease (NAFLD). Increasing evidence is supporting the potential application of natural resourced polysaccharides as promising prevention or treatment strategies against NAFLD. In the current study, an acidic heteropolysaccharide, LFP-a1, was isolated and purified from Lycii fructus with successively hot water refluxing extraction, alcohol precipitation, protein removal, and DEAE-52 cellulose chromatographic separation. LFP-a1 was a complicated structured polysaccharide with an average MW of 4.74 × 104 Da and composed of 6 monosaccharides and 1 uronic acid. Preexposure of LFP-a1 could increase the cell viability and reverse the abnormal oxidative stress though inhibition of mitochondrial-mediated apoptotic pathway and correction of cell cycle progression against H_2_O_2_ hepatoxicity in NAFLD model L02 cells. Consistently, *in vivo* study in thioacetamide- (TAA-) induced NAFLD model zebrafish larvae showed LFP-a1 preserved the liver integrity and alleviated TAA-induced oxidative stress through downregulation of abnormal apoptosis. These observations indicated the hepatoprotective activity of LFP-a1, which may be applied for the prevention or treatment of NAFLD or other oxidative stress-related diseases.

## 1. Introduction

The conception of oxidative stress basically defines a disturbed condition in which accumulative molecular and/or cellular oxidative injury is observed with the disequilibrium of reactive oxygen species (ROS) generation and in-build antioxidant defensing system [1]. Excessive, repetitive, and/or sustained oxidative stress stems from elevated formation and accumulation of reactive oxygen intermediates (ROI, including free radicals, hydrogen peroxide, hydroxyl radicals, and superoxide anion) have been implicated to be closely interconnected with the initiation and progression of a myriad of pathological lesions which often result in metabolic damage such as aging, cardiovascular diseases, neurological disorders, inflammation, and liver disease. ROS accumulation in hepatocyte is regarded as a substantial contributor in the pathogenesis of a hepatic manifestation of metabolic syndrome, nonalcoholic fatty liver disease (NAFLD), ranging from steatosis, steatohepatitis, and cirrhosis to carcinoma [2]. Oxidative stress-mediated toxicity, accompanied by changes in lipid metabolism, gradually destroys the balance between ROS production and detoxification pathways, which further results in feedback overaccumulation of ROS, deficiencies of antioxidant defense, and inevitable impairment of mitochondrial respiratory function in hepatocyte. The excessive and sustained oxidative stress contributes to hepatocellular damage that leads to functional impairments. Considering the constant exposure of liver cells to oxidative stress, scavenging of ROS by antioxidants may provide a persuasive therapeutic strategy targeting at least one key pathological event related to NAFLD, i.e., solemn oxidative damage [3]. Actually, previous research has established the protective or therapeutic potency of natural antioxidants upon oxidative injury induced by nonalcoholic insult [4].

One of the scientific hallmarks of the past decades has been the technologies and methodologies to identify the details of structure of polysaccharides, which accelerate the pace of discovery of polysaccharide functions [5]. The current accomplishments have led to the establishment of polysaccharide-related studies as one of the fundamental areas of biological and biochemical studies, as exemplified by the increasingly recognized spectrum of pharmacological benefits, such as ant-oxidic, antiaging, antimicrobial, anticancer, antithrombotic, hypoglycemic, immunomodulatory, and gut microbial modulatory [6–11]. Emerging innovative research output has evaluated applying natural resourced polysaccharides as promising prevention or treatment strategies against a variety of human disease models [12, 13]. Specially, unlike the currently used synthetic chemical components which were suspected to be associated with hepatic damage resulted from the treatment, natural polysaccharides have been supported by interdisciplinary research to be effective with innate low toxicity and natural compatibility. Applying natural polysaccharides as promising protective agent is a rising concern to protect against chemical-induced hepatic injury.

Lycii fructus, also called goji berry or wolfberry, is the bright reddish orange fruit of solanaceous shrubbery *Lycium barbarum* L. Lycii fructus has long been worldwide consumed as functional dietary tonic in the recipes of worldwide regimen based on the recorded “liver nourishing, lung moistening, and eye lubricating” efficacies in ancient script. In the late years, motivated by the expectations for the promotion of health and longevity, Lycii fructus has been increasingly popular due to its recognized diverse nutritive, preventive, and therapeutic properties including immunoregulation, antioxidation, antiaging, cryoprotection, hepatoprotection, and cancer prevention [14].

Lycii fructus is a rich resource of complex mixture of reported antioxidants, among which are some bioactive small molecules like vitamin C, carotenoids, and polyphenols, which may act in concert to exert activities [15]. Apart from the identified small chemical constituents, Lycii fructus polysaccharides (LFPs) are highlighted by increasing interest to be responsible for its antioxidative capacity, implying that LFPs may be a potential protective or therapeutic agent against liver damage [16]. Therefore, in the present study, we purified LFP fractions from Lycii fructus. In addition, *in vitro* and *in vivo* assays were performed upon human hepatocyte cell line L02 and zebrafish larvae to further our understanding of the latent potentialities and the underlying mechanisms of LFP utilization in protecting liver against oxidative stress in NAFLD.

## 2. Materials and Methods

### 2.1. Chemicals and Materials

The dried fruits of Lycii fructus were purchased from Bairuiyuan Gouqi Corp. (Yinchuan, Ningxia Autonomous Region, P.R. China) and identified by the corresponding author based on the morphological and histological features according to the standards of Chinese Pharmacopoeia (2015 version). Voucher specimen was deposited in Jiangsu Collaborative Innovation Center of Chinese Medicinal Resources Industrialization. DEAE-52 cellulose was purchased from Whatman Ltd. (Kent, UK). Dulbecco's modified Eagle's medium (DMEM) and fetal bovine serum (FBS) were purchased from Life Technologies (Grand Island, NY, USA). Propidium iodide (PI) and Hoechst 33342 were purchased from Sigma–Aldrich (St. Louis, MO, USA). Cell counting kit-8 (CCK-8) and 5-Ethynyl-2'-deoxyuridine (EdU) Imaging Detection Kit were purchased from KeyGEN Biotech Int. (Nanjing, China). Annexin V/fluorescein isothiocyanate (FITC)-PI apoptosis detection kits were purchased from BD Biosciences (Franklin Lakes, NJ, USA). Trizol reagent and reverse transcriptase kits were purchased from Takara Biomecicals Co., Ltd. (Dalian, China). All other chemicals and regents used were of the commercially highest grade unless specifically noted.

### 2.2. Polysaccharide Extraction and Purification from Lycii Fructus

The acidic polysaccharide LFP-a1 was extracted and purified from Lycii fructus with hot water extraction and subsequently purification with ion-exchange chromatography. Briefly, ground Lycii fructus were fluxed with 80% ethanol at 75°C for 2 h followed by acetone-petroleum ether at 55°C for 1 h to remove small lipophilic molecules and pigment. The residue was subjected to two times of reflux water extraction at 90°C for 2 h each time. The polysaccharides were precipitated with ethanol and deproteinated with Sevag reagent (chloroform : butyl alcohol 4 : 1) to give deproteinized LFPs. LFPs were further fractionated on an ion-exchange DEAE-52 cellulose column. After the neutral fractions were removed by water washing, the acidic fraction was eluted with 0.25 M aqueous NaCl and collected based on the chromatography profile at 490 nm determined by phenol-sulfuric acid assay. The collected fraction was concentrated, dialyzed, and freeze dried given a purified fraction LFP-a1, which was subjected to further structural elucidation and activity evaluation.

### 2.3. Primary Structure and Microstructure Characterization of LFP-a1

#### 2.3.1. General Analysis

Carbohydrate content and protein content were measured by phenol-sulfuric acid method and Bradford method using galactose and bovine serum albumin as standard, respectively [17, 18]. Congo red test was used to detect the presence of triple-helical conformation [19].

#### 2.3.2. FT-IR Spectroscopy

LFP-a1 sample was subjected to a Thermo Nicolet iS5 IR spectrometer (Thermo Scientific, USA) to record FT-IR spectrum within the range of 4000–400 cm^−1^ to investigate the vibrations of molecules and polar bonds between the different atoms.

#### 2.3.3. Molecular Weight Distribution of LFP-a1

High performance gel permeation chromatography (HPGPC) was used to visualize the homogeneity and MW distribution on an ELEOS System (Wyatt Technology Co., Santa Barbara, CA, USA) equipped with RID-10A refractive index detector and an OHpak SB-802.5HQ column (8 mm × 300 mm, Showa Denko Co., Tokyo, Japan). Sample was resolved in 0.1 M NaNO3 and eluted at a flow rate of 0.5 mL/min. MW distribution was calculated with reference to the standard curve prepared with T-series Dextran.

#### 2.3.4. Monosaccharide and Uronic Acid Composition Assays of LFP-a1

GC-MS-based monosaccharide and uronic acid composition assays were performed after LFP-a1 sample was hydrolyzed in 2 mL of 2 M TFA at 110°C for 2 h and derived into alditol acetates or N-propylaldonamlde acetates according to the method of Lehrfeld [20] and loaded on an Agilient 7000C GC/MS Triple Quard system (Agilent Technologies, Santa Clara, CA, USA) equipped with an Agilent HP5-ms capillary column (30 m × 0.25 mm × 0.25 *μ*m). Oven temperature was programmed as follows: 100°C for 3 min, then to 200°C at 20°C/min and 200°C for 2 min, to 230°C at 5°C/min and 230°C for 2 min; 280°C at 10°C/min 280°C for 8 min. Data was analyzed with Agilent MassHunter Workstation.

### 2.4. Hepatoprotection of LFP-a1 In Vitro

#### 2.4.1. Cell Line and Maintenance

The human hepatocyte cell line L02 was purchased from Shanghai Cell Bank of Chinese Academy of Science (Shanghai, China). Cells were maintained as mycoplasma-free subconfluent monolayer cultures in DMEM supplemented with 10% inactivated FBS at 37°C in a humidified 5% CO_2_ incubator. In order to evaluate the cytoprotective effects of LFP-a1 in a model of oxidative stress in cultured cells, the following indices were measured: (1) cell viability (CCK8) and cell proliferation (EdU assay), (2) indices of cellular oxidative stress and antioxidant markers, (3) cell apoptosis, and (4) cell cycle distribution.

#### 2.4.2. Cell Viability Assay and Cell Proliferation Assay

L02 cells were seeded at a density of 1 × 104 cells/cm^2^ in triplicate in 96-well plates and allowed to attach before treatment. Cells were them exposed to a series of concentrations of LFP-a1 (0.5, 1, 2 mg/mL) for 24 h and H_2_O_2_ (40, 70, 100 *μ*M) for 24 h separately or successively. Cell viability was then determined with CCK8 assay that shows the mitochondrial activity of living cells with the assistance of microplate spectrophotometer (Infinite 200 PRO, TECAN Inc. Switzer). Cell viability index was expressed as percentage of vehicle control (cells only suffered medium change) normalized to 100%. In addition, cell proliferation was evaluated with cells seeded in 6-well plates by EdU imaging detection. Briefly, cells were treated as described above, then 10 *μ*M EdU was added for 2 h before the end of the culture to label proliferating cells. After being fixed by 4% paraformaldehyde and added by Click-iT reaction cocktail, cells were subjected to Hoechst 33342 for nuclei counterstaining. Cell proliferation, expressed as percentage of EdU positive cells to all cells, was achieved by the EdU- and Hoechst 33342-stained cells in the overlay photographs of five random visual fields in each well under a fluorescent microsystem (Leica DMI3000B, Wetzlar, Germany) facilitated with LAS program. Quantification was performed with image J software (https://imagej.nih.gov/ij/).

#### 2.4.3. Biochemical Determination of Hepatic Oxidative Stress and Antioxidant Indicators

L02 cells seeded in 96-well plates were cultured and pretreated with LFP-A1 for 24 h, after which cells were challenged with 70 *μ*M of H_2_O_2_ for another 24 h as described in Section 2.4.2. Intracellular ROS and oxidative stress (NO, MDA levels as oxidative damage markers, activities of SOD, GSH-Px, and CAT, GSH level, and T-AOC as antioxidant markers) were assessed with commercially available assay kits (Jiancheng Bioengineering Int. Nanjing, China) by strictly following the manufacturer's instructions (Abbreviations: NO, nitric oxide; MDA, malondialchehyche; SOD, superoxide dismutase; GSH-Px, glutathione peroxidase; CAT, catalase; GSH, reduced glutathione, T-AOC, total antioxidant capacity).

#### 2.4.4. Cell Apoptosis Analysis

Apoptosis was first detected with fluorescent Hoechst 33342-assisted nuclear staining. Briefly, cells were cultured and treated as described in Section 2.4.3, then washed and incubated with 10 *μ*g/mL of Hoechst 33342 for 20 min at 37°C in dark. Apoptosis was observed by photographing the cells of five random visual fields in each well.

Apoptosis was further quantitatively detected with Annexin V-FITC/PI double staining in conjunction with flow cytometry. L02 cells were seeded and cultured in 25 ml flasks and treated as described in Section 2.4.3. Cells were then washed, harvested, resuspended, and double-stained with 2.5 mg/ml Annexin V-FITC for 15 min and 1 mg/ml PI for 10 min in dark. DNA histograms were obtained by fluorescence activated cell sorting analysis (FAQS) on a BD AccuriC6 Cytometer (BD Biosciences, Franklin Lakes, NJ, USA) affiliated with BD FACSuite Flow Cytometry software. For each analysis, at least 10,000 gated events were collected. Percentage of Annexin V-positive cells gated in the PI-negative/positive fractions was calculated as the percentage of cells in early/late apoptosis, respectively.

#### 2.4.5. Western Blot Analysis

Proteins were extracted from cell pellets in ice-cold lysis buffer. After protein concentrations determined, equal amounts of total protein (30 *μ*g) of each sample were fractionated on 10% SDS-PAGE gel, electro-transferred onto 0.2 *μ*m nitrocellulose membrane, and immune-blotted with diluted corresponding primary antibodies overnight at 4°C followed by horseradish peroxidase-conjugated secondary antibodies for 1 hour at 25°C. Chemiluminescence signal was visualized and recorded with the ChemiDoc™ Touch Imaging System (Bio-Rad Laboratories, Inc., USA).

#### 2.4.6. Cell Cycle Distribution Analysis

Cell cycle distribution analysis was performed using flow cytometry on the same BD AccuriC6 Cytometer. After the corresponding treatment period of LFP-a1 and H_2_O_2_ as described in Section 2.4.3, both adherent and detached cells were collected and fixed with ice cold 70% ethanol overnight. Cells were resuspended in 1 mL staining solution (PBS containing 0.1% Triton X-100, 10 *μ*g/mL DNase-free RNase and 50 *μ*g/mL PI) for 30 min in dark. At least 30,000 gated events were collected to permit each cell cycle analysis. Histograms were analyzed using Flow-Jo version 10.0 (Tree Star, Ashland, OR, USA).

### 2.5. Hepatoprotection of LFP-a1 In Vivo

#### 2.5.1. Zebrafish Strains and Husbandry

Zebrafish experiment was approved by the Animal Care and Use Committee of Nanjing University of Chinese Medicine. Wild-type zebrafish (AB strain) and a transgenic line of zebrafish with enhanced liver-specific green fluorescent protein (eGFP) expression [Tg(*lfabp10a-eGFP*)] were purchased from Xinjia Zebrafish Resource and Screening Platform (Nanjing, China). Adult zebrafish were kept in an automatic recirculating tank at a density of 1.5 fish per liter at the following conditions: pH 7.5-8.0, dissolved oxygen 7.5 mg/L 28.5°C, and constant 14/10 h light/dark cycle. Newly fertilized eggs were collected from natural spawning within 30 min and randomly sorted into 6-well culture plates containing embryo medium and kept in a 28.5°C incubator controlled in 14/10 h light/dark cycle.

#### 2.5.2. Waterborne Exposure of Zebrafish to LFP-a1 and Thioacetamide (TAA)

3 dpf larvae in normal development were randomly allocated into five groups and transferred into 12-well plate (10 larvae in a well, ~100 larvae for one group). Larvae were exposed to different concentrations of LFP-a1 (0, 0.2, 0.5, and 1 mg/mL) for three days, followed by 7 mM TAA insult for another 3 d. Data were expressed as a percentage of vehicle control (larvae only suffered medium change) normalized to 100%.

#### 2.5.3. Fluorescence Image Acquisition

The liver green larvae after treatment were anesthetized with 0.16% (*w*/*v*) tricaine, mounted with 3% methylcellulose, and imaged in bright and fluorescent field under a Leica stereo microsystem to observe the liver phenotype in a lateral view. The size and fluorescence intensity were obtained by Image J.

#### 2.5.4. Transaminase Analysis

For the hepatic transaminase activity analysis, the AB strain larvae were collected, anesthetized, and homogenized in 400 *μ*L ice-cold saline. The homogenate was centrifuged at 15,000×g for 15 min at 4°C to remove debris. The supernatant was then analyzed for alanine transaminase (ALT) and aspartate transaminase (AST) activities using the commercial spectrophotometric diagnostic kits (Jiancheng Bioengineering Int. Nanjing, China).

#### 2.5.5. Whole-Mount *Oil Red O Staining*

Larvae after treatment were fixed in 4% paraformaldehyde at 4°C overnight and washed twice in phosphate-buffered saline containing 0.1% Tween-20, followed by 0.5% Oil Red O working solution staining in dark for 30 min. Samples were then rinsed in successive 80%, 40%, and 20% propylene glycol to remove nonspecific staining. Images were obtained in bright field under a Leica stereo microsystem to observe the liver phenotype in a lateral view. The size and mean gray value were quantified by Image J to quantitatively evaluate hepatocyte steatosis.

#### 2.5.6. Oxidative Stress and Antioxidant Indicators Determination

To evaluate the protective effect of LFP-a1 against TAA-induced oxidative stress, the AB strain larvae were subjected to oxidative stress measure. Larvae were anesthetized and homogenized in 400 *μ*L ice-cold saline. The homogenate was centrifuged at 15,000×g for 15 min at 4°C to remove debris. The supernatant was then subjected to determine the biomarker of oxidative stress as described in 2.4.3.

#### 2.5.7. RNA Extraction and Real-Time Quantitative PCR (qPCR)

Total RNA was isolated from larvae using TRIzol reagent. Approximately 1 *μ*g of RNA was reverse-transcribed into cDNA using PrimeScript RT Master Mix. qPCR application was performed in a total volume of 20 *μ*L containing 10 *μ*L SYBR Green One-Step RT-PCR Master Mix and 1 *μ*L cDNA template on a real-time quantification system (LightCycler 96, Roche, Switzerland). Quantification was analyzed using the comparative Ct relative quantification method formula 2^−ΔΔCT^, with housekeeping gene GADPH mRNA used as invariant control to normalize the mRNA of target genes. PCR applications were performed in triplicate. Specific primers summarized in Table S1 were used in this study.

### 2.6. Statistical Analysis

Data results were presented as mean ± standard deviation (SD) of at least three independent experiments. Differences were considered statistically significant once *p* values were less than 0.05 using one-way analysis of overall variance (ANOVA) followed by Tukey's multiple comparison tests. All statistical analyses were performed using GraphPad Prism software 8 project for Windows (GraphPad Software, San Diego, CA, USA).

## 3. Results and Discussion

### 3.1. Extraction, Purification, and Physicochemical Properties of LFP-a1

**Figure 1:**
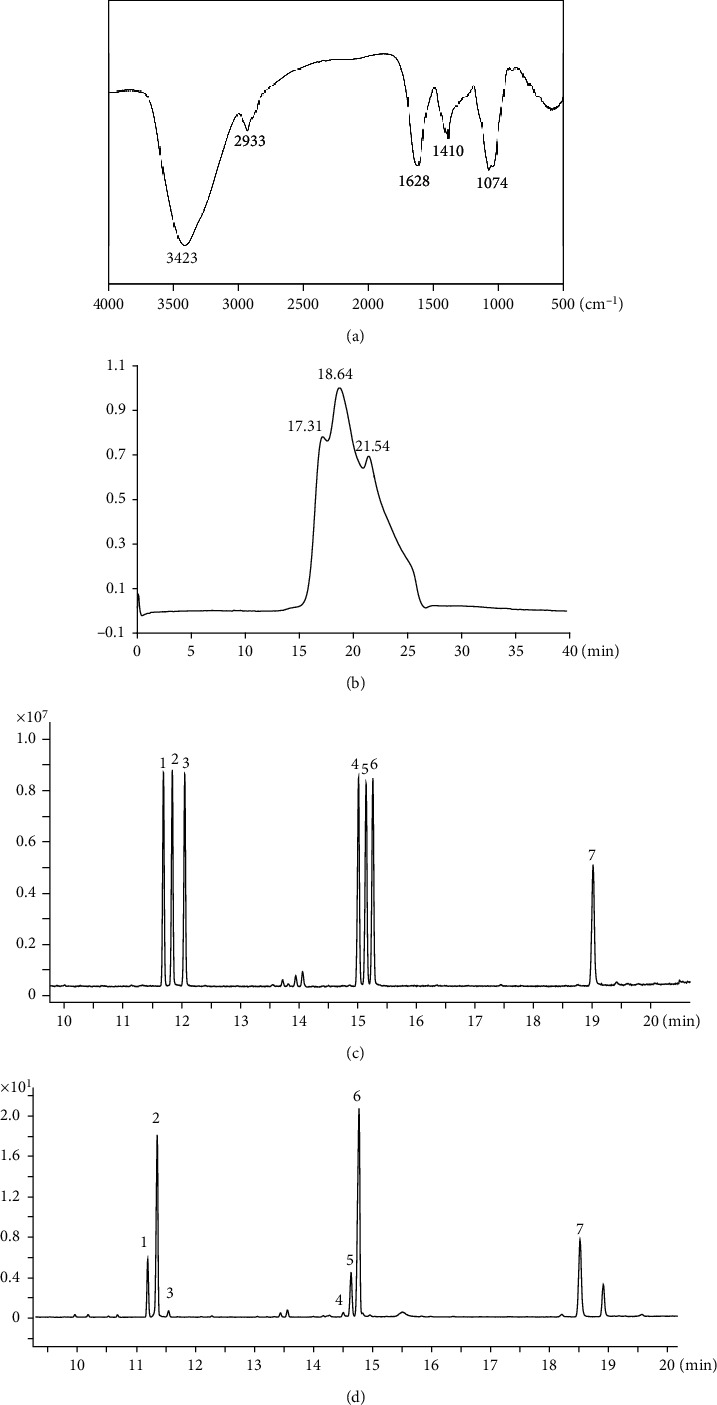
Structural analysis of LFP-a1. (a) FT-IR spectrum; (b) HPGPC profile on OHpak SB-802.5HQ column chromatographic columns; (c, d) GC-MS-based monosaccharide and uronic acid composition of (c) mixed standard and (d) LFP-a1. Peaks: (1) rhamnose, (2) arabinose, (3) xylose, (4) mannose, (5) glucose, (6) galactose, and (7) galacturonic acid.

Water soluble deproteinized LFPs were successfully isolated from Lycii fructus powder by a series of procedures including ethanol infusion, water extraction, ethanol precipitation, and deproteination. After subsequent purification by DEAE-cellulose, an acidic polysaccharide LFP-a1 was finally achieved for further evaluation. After being lyophilized, LFP-a1 was fluffy and yellowish in color. The contents of total carbohydrate and protein of LFP-a1 were estimated to be 87.43% and 3.12%, respectively. LFP-a1 was soluble in water and practically insoluble in acetone, ethanol, chloroform, diethyl ether, and ethyl acetate. LFP-a1 showed negative iodine-potassium iodide reactions, indicating the absence of starch type polysaccharide. No red shift of maximum absorption was observed at any concentrations in Congo red test, indicating that LFP-a1 did not form a triple-helical conformation in solution. Infrared spectroscopy ranged of 400-4000 cm^−1^ exhibited typical absorption peaks assigned to saccharide matrix as shown in Figure 1(a) and summarized in Table 1. Monitored by HPGPC, the MW distribution profile of LFP-a1 showed a dominated continuous peak accompanied with a minor component of higher MW and a lower MW, indicating the polydisperse nature of LFP-a1 (Figure 1(b)). The weight-average MW of the dominated peak was 4.74 × 104 Da as calculated by the calibration curve. Meanwhile, LFP-a1 was composed of rhamnose, arabinose, xylose, mannose, glucose, galactose, and galacturonic acid in molar ratio of 8.87 : 37.17 : 0.61 : 0.52 : 6.84 : 37.20 : 8.79, indicating a complicated composition of LFP-a1 (Figures 1(c) and 1(d)).

### 3.2. Hepatoprotection of LFP-a1 on H_2_O_2_- Insulted NAFLD Model L02 Cells *In Vitro*

#### 3.2.1. LFP-a1 Attenuated Cytotoxicity Induced by H_2_O_2_ in L02 Cells

**Figure 2:**
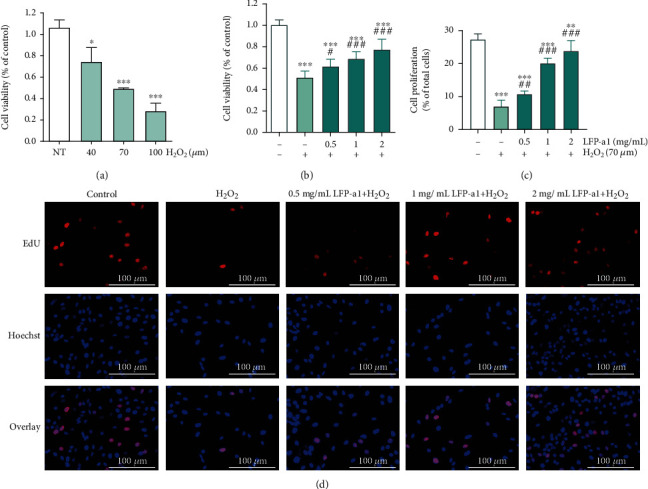
Cytotoxicity of H_2_O_2_ and protective effects of LFP-a1 in H_2_O_2_-challenged L02 cells. (a) Effect of H_2_O_2_ on cell viability of L02 cells, respectively. (b) Effect of LFP-a1 on cell viability of L02 cells against H_2_O_2_-insult. (c) Effect of LFP-a1 on cell proliferation of L02 cells against H_2_O_2_-insult. (d) Edu and Hoechst 33342 fluorescent staining of L02 cells (EdU: staining in red; Hoechst 33342: staining in blue, original magnification: ×200). Data are expressed as the mean ± SD (*n* = 3) and are representative of at least three independent experiments (^∗^*p* < 0.05, ^∗∗^*p* < 0.01, ^∗∗∗^*p* < 0.001 compared with control cells, ^**#**^*p* < 0.05, ^**##**^*p* < 0.01 compared with H_2_O_2_-challenged cells).

As a strong chemical factor of oxidants, H_2_O_2_ can penetrate hepatic cell membrane easily into cell nucleus without being enzymatic degraded and trigger complex hepatic disorder caused by overdose ingestion of toxin. One of the pathological mechanisms has been demonstrated to be the involvement of oxidative stress. Nowadays, this H_2_O_2_-trigged cell model has been widely used as an *in vitro* cellular screening platform to investigate hepatotoxicity and hepatoprotection efficacy. Hereby, L02 cells were subjected to exogenous addition of LFP-a1 and/or H_2_O_2_ to address the potential cytoprotection of LFP-a1 against oxidative stress-induced hepatotoxicity. Colorimetric cck-8 assay and Edu imaging assay were performed to measure the mitochondrial metabolic rate of growing cells as an indirect value of cell viability and cell proliferation, respectively. Different concentrations of LFP-a1 (ranging from 0 to 2 mg/mL) exposure induced negligible viability loss with respect to vehicle control L02 cells (data not shown). In sharp contrast, as depicted in Figure 2(a), H_2_O_2_ induced significant inhibition of cell survival in a dose-dependent manner. When cells were pretreated with LFP-a1 for 24 h, however, cell viability was gradually restored to 61.6% at 0.5 mg/mL and 71.3% at 2 mg/mL of LFP-a1, respectively. Similarly, as shown in Figures 2(c) and 2(d), exposure of H_2_O_2_ seriously inhibited L02 proliferation. Pretreatment with LFP-a1 progressively increased the cell proliferation to 10.74% at 0.5 mg/mL and 23.81% at 2 mg/mL of LFP-1, respectively, as evidenced by the increased number of viable EdU-positive cells. This pattern of cell viability and cell proliferation ambiguously indicated that LFP-a1 had protective potentials by significantly attenuating H_2_O_2_-induced cytotoxicity in L02 cells.

#### 3.2.2. LFP-a1 Mitigated H_2_O_2_-Induced Oxidative Stress Scenario

ROS generation during metabolism in normal physiological-steady state is compensated for by the endogenous antioxidant system. However, excess ROS accumulation can result in oxidative stress, which was believed to be one of the real culprits responsible for the progressive decline of cell health and for the consequent structural and functional alteration [21, 22]. ROS production was consequently been considered as a routine index of overall oxidative stress in living cells [23]. Uncharged NO concentration increased greatly upon reaction with superoxide radical, serving as an important intra- and intercellular messenger in oxidative stress [24]. Current therapeutic strategies for NAFLD majorly focused on the reduction of hepatic lipid overaccumulation. Based on the fact that lipid metabolism was closely related oxidative stress, MDA, one of the important end products of lipid peroxidation triggered by ROS, was identified to be a crucial biomarker reflecting oxidative damage to molecules [25]. In addition, SOD, GSH-Px, and CAT collectively constitute a line of a cellular antioxidant enzyme in the endogenous antioxidant defense system involved in ROS scavenging by converting superoxide and removing peroxide [26]. GSH, a tripeptide that works by interrupting free radical chain reactions [27], works as a nonenzymatic mechanism with the enzymes in a mutually supportive way to provide a protective defense against ROS. When the ROS increased to an extent beyond the capacity of the endogenous defense system, oxidative stress may be caused. The development and treatment of oxidative-stress was usually described by measurement of T-AOC, an indicative of the cellular ability to counteract oxidative stress-related damage.

**Figure 3:**
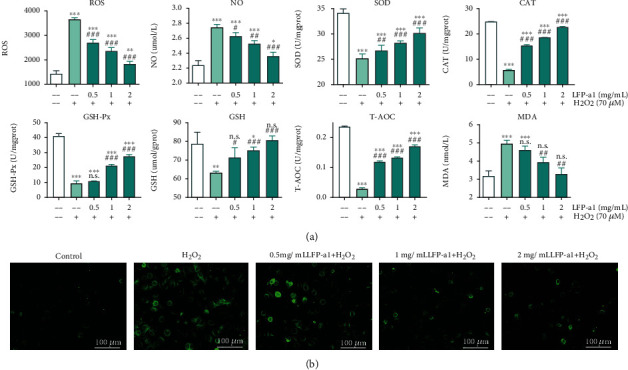
LFP-a1 mitigated H_2_O_2_-induced oxidative stress scenario. (a) Effect of LFP-a1 on ROS production and oxidative stress indicators in H_2_O_2_-challenged L02 cells. (b) ROS detection images of L02 cells. Cells were treated with different concentrations of LFP-a1 and 70 *μ*M H_2_O_2_ successively. Data were presented as mean (*n* = 3) ± SD of three independent experiments (^∗^*p* < 0.05, ^∗∗^*p* < 0.01, ∗∗∗*p* < 0.001 compared with H_2_O_2_ groups, ^**#**^*p* < 0.05, ^**##**^*p* < 0.01, ^**###**^*p* < 0.001 compared with H_2_O_2_-challenged cells).

Increasing lines of evidence proved that oxidant H_2_O_2_ induces acute NAFLD-like oxidative stress in different kinds of cells. This cell model has provided a useful instrument for clarifying the underlying mechanism and probing possible treatment for NAFLD in the fields of hepatology [28]. Investigating the effects of LFP-a1 upon oxidative stress in L02 cells could provide more direct evidence for its potential application. Therefore, the variation of intracellular ROS production and indices interpreting oxidative stress were tracked and measured to assess the protective activity of LFP-a1 against oxidative stress in H_2_O_2_-challenged L02 cells (Figure 3).

As illustrated in Figure 3, considerable overproduction of intracellular ROS, NO production, and MDA formation were observed in H_2_O_2_-challenged groups, accompanied by loss of both enzymatic (SOD, GSH-Px, and CAT activities) and nonenzymatic (GSH levels) mechanism, indicating disturbance in the normal physiological redox state of L02 cells under H_2_O_2_ insult. On top of this disturbance, these oxidative stress indices could be dose-dependently recovered with LFP-a1 pretreatment, as characterized by the alleviated intracellular ROS production, NO production, MDA generation, and renewed endogenous SOD activity, GSH-Px activity, CAT activity, and GSH level. Consistently, measurement of T-AOC provided similar scenario. The trend of changing observed was correlated with the suppression of intensified ROS-induced oxidative stress in L02 cells. These events collectively pointed to the suggestion that upstream LFP-a1 treatment was able to specifically reverse the abnormal state of ROS release, lipid peroxidation damage, and inner antioxidants activity, indicating that LFP-a1 had a wide scope to sequester ROS involved to prevent oxidative stress. Inhibition of ROS production could be one of the mechanisms for the cytoprotection of H_2_O_2_ challenged L02 cells.

#### 3.2.3. LFP-a1 Impacted Apoptosis Induction and Cell Cycle Progression in H_2_O_2_-Challenged L02 Cells

**Figure 4:**
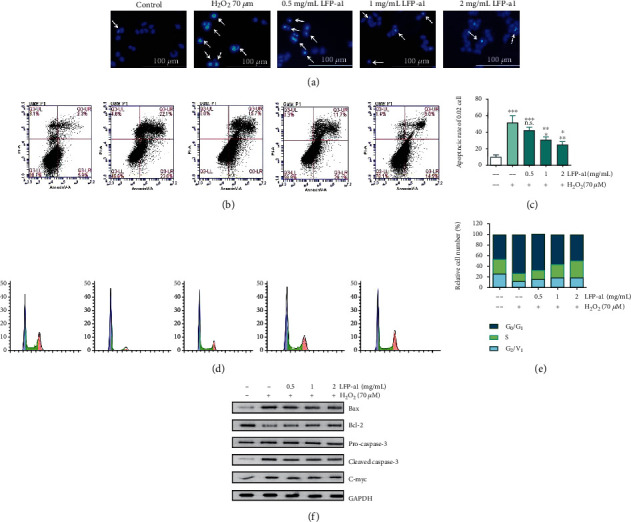
Effects of LFP-a1 on cell apoptosis and cell cycle in H_2_O_2_-challenged L02 cells. Cells were pretreated with different concentrations (0.5, 1.0, and 2 mg/mL) of LFP-a1 for 24 h prior to incubation with 70 *μ*M H_2_O_2_ for an extra 24 h. (a) Representative fluorescent images of cells stained with Hoechst 33342 and observed under a fluorescent microscope; (b) flow cytometry analysis of apoptosis after double-staining with Annexin V-FITC/PI; (c) bar graphs of apoptotic ratio of cells analyzed by flow cytometry; (d) representative DNA histograms of cell cycle distribution after staining with PI; (e) statistical results of population in different cell cycle phases analyzed by flow cytometry; (f) protein expressions of apoptosis-associated biomarkers in L02 cells measured by Western blot. Results were expressed as means ± SD (*n* = 3) and representative of at least three separate experiments. ^∗^*p* < 0.05, ^∗∗^*p* < 0.01, ^∗∗∗^*p* < 0.001 compared with control cells, ^**#**^*p* < 0.05, ^**##**^*p* < 0.01, ^**###**^*p* < 0.001 compared with H_2_O_2_-challenged cells.

Cell apoptosis is a cascade process of programmed cell death viewed as a leading pathway for degenerative disorders [29]. The relation between stress and cell apoptosis has attracted much attention. In addition, cell cycle distribution is of valuable interpretation of changes in cell proliferation and apoptosis [30]. Therefore, induction of apoptosis and cell cycle distribution were detected to reveal whether the apoptotic like mechanism and cell cycle were associated with cytoprotective effect of LFP-a1. Oxidative stress-induced alterations in apoptosis and cell cycle distribution in L02 cell cultures preincubated with LFP-a1 with H_2_O_2_-triggered oxidative stress are shown in Figure 4.

Figure 4(a) illustrated the variation of nuclear morphology with response to the exogenous LFP-a1 and H_2_O_2_ insult. To this feature, cells with homogeneously chromatin distribution were considered viable, whereas the presence of chromatin condensation and/or nuclear fragmentation was indicative hallmarks of apoptosis. To confirm and assess the proportion of apoptotic cells, cells were further subjected to Annexin-V/PI staining followed by flow cytometry, in which apoptotic cells were expressed as Annexin V positive DNA fragmentation (Figure 4(b)). Based on the profound accumulation of chromatin condensation and/or nuclear fragmentation in Hoechst 33342 staining-assisted morphology and Annexin V+ cells in flow cytometric diagram, H_2_O_2_ insult remarkably facilitated apoptosis in L02 cells to ~50% with respect to the basic level of apoptosis in control group. When preprotected with different concentrations of LFP-a1, however, the abnormal apoptosis progressively decreased to 42.05%, 30.75%, and 24.77%, respectively (Figures 4(b) and 4(c)), indicating that in this context, LFP-a1 gradually obliterated the occurrence of H_2_O_2_-induced apoptotic death in L02 cells in a dose-dependent manner.

Fidelity of sequential transition from one phase of to another is of great significance to maintain smooth cell cycle progress. Cells must pass through G_0_/G_1_, S, and G_2_/M phases sequentially to complete a cycle. Dysfunction of cell cycle regulation is in close connection with, to some extent, the initiation and progression of disorders originated from external injurious factors [31]. In addition to the established role in promoting cell survival and proliferation, ROS can also trigger cell cycle arrest. We thus sought to assess LFP-a1-mediated cell cycle progression in H_2_O_2_-challenged cells by analyzing the percentage of cells in the G_0_/G_1_, S, and G_2_/M phases (Figures 4(d) and 4(e)). Cells were subjected to PI staining followed by flow cytometric cell cycle analysis for cellular DNA content. Inspection of cell cycle analysis data for L02 cells demonstrated notorious enrichment of G_0_/G_1_ phase cell populations accompanied by decrement of S phase and G_2_/M phase cell populations in H_2_O_2_ group, indicating DNA damage during S phase, or unrepaired damage of certain proteins progressing into next phase. When protected with LFP-a1, G_0_/G_1_ phase population stepwise dropped with the increase of LFP-a1. Commensurately, cells in S phase climbed from 18.0% to 30.9%. This pattern of cell cycle indicated while H_2_O_2_ challenging meditated cell cycle progression arrest at G_0_/G_1_ phase to S phase transition, exogenous LFP-a1 was able to accelerate cells passing into S phase from G_0_/G_1_ phase to restart the cell cycle, which further promoted regeneration of L02 cells in spite of H_2_O_2_ stimuli.

Imbalance between the proapoptotic Bax and the antiapoptotic Bcl-2 could affect cell death [32]. Therefore, possible modulation in the Bax and Bcl-2 expression upon LFP-a1 pretreatment was investigated to achieve the visualized blots of apoptosis-related protein expression. As shown in Figure 4(f), the Bax/Bcl-2 expressions underwent a significant increase/decrease in L02 cells under H_2_O_2_ stimuli. LFP-a1 pretreatment, however, effectively reversed the imbalance between Bax and Bcl-2 compared with H_2_O_2_ damaged cells. Consistently, the increased expression upon H_2_O_2_ insult of activated caspase-3 protein, a key downstream effector and the final apoptotic executioner, was significantly blocked by LFP-a1 pretreatment, albeit no significant variation in full-length pro-caspase-3 expression was observed. The expression of C-Myc, another marker molecule of mitochondrial damage, was curtailed obviously by H_2_O_2_ and elevated gradually by LFP-a1 pretreatment. It can be explained that the hepatoprotective effects of LFP-a1 against H_2_O_2_-induced toxicity may be attributed, at least in part, to the inhibition of mitochondrial-mediated apoptotic pathway. Elucidating research is still necessary to look further inside the full spectrum of detailed mechanisms underlying.

### 3.3. Hepatoprotection of LFP-a1 on TAA-Insulted NAFLD Model Larval Zebrafish In Vivo

#### 3.3.1. Protective Effect of LFP-a1 on TAA-Induced Hepatic Damage in Larval Zebrafish NAFLD Model

**Figure 5:**
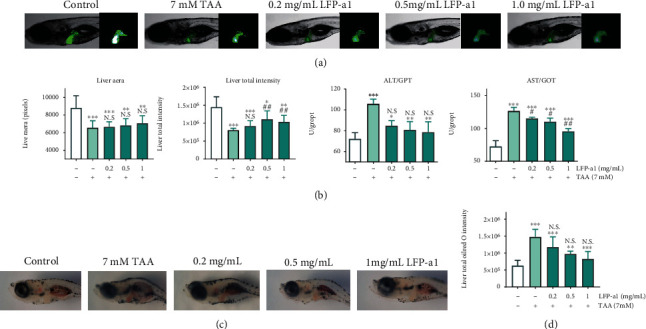
Effects of LFP-a1 on liver development and function in TAA-damaged zebrafish larvae. (a) Effects of LFP-a1 on TAA-induced morphological alterations in larvae livers (×2.5 magnification); (b) statistical effects of LFP-a1 on TAA- induced zebrafish larvae liver area, fluorescence intensity, and ALT and AST activities; (c) images of LFP-a1 on lipid accumulation in larvae livers; (d) statistical effects of LFP-a1 on lipid accumulation in larvae livers (^∗^*p* < 0.05, ^∗∗^*p* < 0.01, ^∗∗∗^*p* < 0.001 compared with control larvae, ^**#**^*p* < 0.05, ^**##**^*p* < 0.01, ^**###**^*p* < 0.001 compared with TAA-challenged larvae, N.S.: no significance).

Zebrafish (Danio rerio) has emerged as an advantageous *in vivo* vertebrate model organism for mechanistic interpreting and high-throughput candidate drug screenings. Zebrafish offers a combination of rapid development, high fecundity, high-throughput screening, and significant morphological and physiological homology to mammals in spite of the reasonable cost [33, 34]. Specially, zebrafish completes primary liver morphogenesis in 48 h postfertilization (hpf) and full liver function in 72 hpf. The quick liver development and its high conservativeness made zebrafish a universal model organism for hepatotoxicity and hepatoprotection screening, basic understanding of acquired liver diseases [35]. Hereby, to further investigate antioxidant activities of LFP-a1 and its potential application in NAFLD treatment, larval zebrafish were exposed to exogenous addition of LFP-a1 in TAA-induced NAFLD model. 3 dpf larvae were pretreated with different concentrations of LFP-a1 and then damaged by 7 mM TAA. Specially, the transgenic line of zebrafish with enhanced liver fluorescence was used to visualize the liver location and the degree of liver damage. As displayed in Figures 5(a) and 5(b), both liver area and total intensity of larvae were reduced as results of 7 mM TAA insult, indicating significant liver developmental toxicity. Pretreatment with LFP-a1, however, gradually increases the liver area (although not statistically significant) and total fluorescence intensity. In addition, larvae liver gradually regained characteristic morphology with increased concentration of LFP-a1, offering ameliorated phenotypic disorders of liver injuries. Furthermore, transaminase activities of cytosolic liver marker enzymes, ALT and AST, were significantly elevated following TAA exposure. Pretreatment of LFP-a1 significantly prevented TAA-induced hepatotoxicity, as evidenced by the decreased activities of ALT and AST, suggesting that LFP-a1 attenuated the hepatic dysfunction or degeneration resulted from exogenous TAA application. Similarly, as shown in Figure 5(c), abnormal abundant lipid accumulation characterized with the increased carmine intensity was observed in the larvae after TAA exposure. However, the intensity was gradually decreased upon LFP-a1 pretreatment, indicating LFP-a1 reduced the hepatic lipid accumulation on larval zebrafish. These results suggested that LFP-a1 feeding effectively preserved the liver integrity, reversed TAA-caused liver damage, and lipid accumulation in larval zebrafish.

#### 3.3.2. Effect of LFP-a1 against Oxidative Stress in TAA-Damaged Larval Zebrafish NAFLD Model

**Figure 6:**
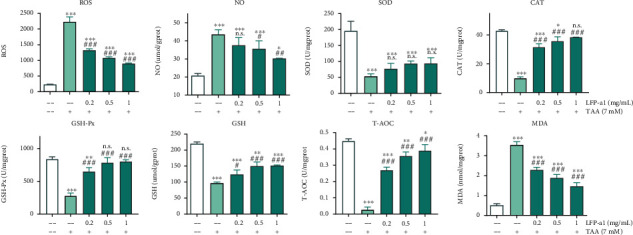
LFP-a1 protected zebrafish larvae from TAA-induced oxidative stress (ROS production, NO production, antioxidant enzyme activities of SOD, GSH-Px, and CAT, GSH content, T-AOC, and MDA level). Larvae were treated with different concentrations of LFP-a1 and 7 mM TAA successively. Data were presented as mean (*n* = 3) ± SD of three independent experiments (^∗^*p* < 0.05, ^∗∗^*p* < 0.01, ^∗∗∗^*p* < 0.001 compared with control, ^**#**^*p* < 0.05, ^**##**^*p* < 0.01, ^**###**^*p* < 0.001 compared with TAA-challenged larvae, N.S.: no significance).

Redox homeostasis is pivotal components for maintenance of normal physiological stability. Injury may occur at high level of oxidative stress when systematic adaptation is not adequate for the stockpile of oxidation products. Lipid peroxidation is the downstream consequence of ROS-mediated damage of membrane lipids. To evaluate the protective effect of LFP-a1 against oxidative stress scenario, ROS production and other oxidative stress indices were estimated in TAA-induced larval zebrafish NAFLD model. The biochemical analysis results provided evidence that oxidative stress occurred due to the 3 d TAA insult as shown by the quantified histograms in Figure 6(a) and representative fluorescence images in Figure 6(b). Compared with the basal level of oxidative stress in physiological status, the imposed TAA initiated deleterious reduction-oxidation disequilibration seen by the increased level of ROS and NO, weakened enzymic defense system (SOD, CAT, and GSH-Px activities), reduced GSH levels and T-AOC, suggesting depletion of antioxidant defense system, and impaired elimination of xenobiotics metabolites. As a result, increased lipid peroxidation was detected in TAA-intoxicated larvae liver. However, the degree of ROS production and disequilibration were obviously ameliorated as a response to the exogenous LFP-a1 feeding. The overproduction of ROS was decreased, accompanying the emergence of the rescued NO concentration and reactivated enzymic defensing. Not unexpectedly, the improved endogenous antioxidative network consequently enhanced the downstream lipid metabolism, as indicated by the decreased level of MDA. These results clearly indicated the effective protection of LFP-a1 pretreatment against TAA-induced oxidative stress in larval zebrafish NAFLD model.

#### 3.3.3. Effects of LFP-a1 on Apoptosis-Related Gene Expression of Zebrafish Larvae with NAFLD

**Figure 7:**
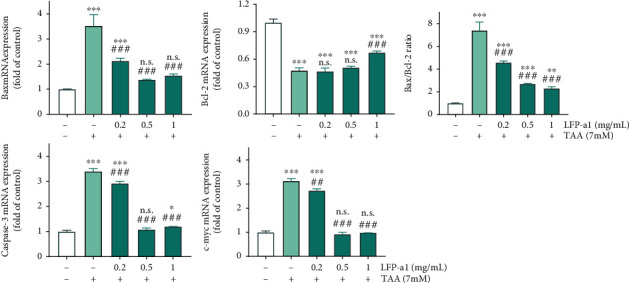
mRNA levels of apoptosis-related gene in larval zebrafish NAFLD model measured by qPCR. Larvae were treated with different concentrations of LFP-a1 and 7 mM TAA successively. Data were presented as mean (*n* = 3) ± SD of three independent experiments (^∗^*p* < 0.05, ^∗∗^*p* < 0.01, ^∗∗∗^*p* < 0.001 compared with control, ^**#**^*p* < 0.05, ^**##**^*p* < 0.01, ^**###**^*p* < 0.001 compared with TAA-challenged larvae, N.S.: no significance).

The mRNA expression levels of apoptosis-related factors Bax, Bcl-2, caspase 3, and c-myc were quantitatively analyzed by qPCR amplification to detect DNA damage initiated from etiologic oxidative stress. Consistently with the *in vitro* assessment, profound ROS accumulation, and depotentiation of ROS scavengers increased oxidative stress, which then initiated apoptotic processes. Luckily, the significant disorganization of Bax/Bcl-2 ratio initiated by TAA damage was as downmodulated by LFP-a1 as shown in Figure 7. Specially, when carefully analyzing the overall changing features, this downmodulation was mainly due to an inhibition of Bax expression rather than an increase in Bcl-2 mRNA levels. The changing pattern of mRNA expressions of caspase-3 and c-myc were observed to be parallel very closely. They both underwent a sharp increase in TAA-challenged larvae, which were restored by LFP-a1 pretreatment, supporting the importance of LFP-a1for inhibiting apoptosis in NAFLD larvae. These results correlated with the alleviated hepatic damage and oxidative stress scenario observed in H_2_O_2_-induced NAFLD model L02 cells *in vitro* as discussed above.

The targets of hepatic damage triggered by 7 mM TAA were heterogeneously complicated, involving a broad spectrum from the phenotypic and functional changes in the development process, the emergence of disorganization of antioxidant system, and the consequent apoptosis induction, to the metabolic imbalance. Pretreatment of LFP-a1 preserved the liver integrity and showed marked protective effect upon the liver function, terminating the overproduction of ROS that escaped the antioxidant defenses through reorganization of the antioxidant system. LFP-a1 feeding alleviated TAA-caused DNA damage through downregulation of abnormal apoptosis, further suggesting the hepatoprotective effect and potential application of LFP-a1 in the prevention and treatment of NAFLD.

## 4. Conclusions

In the present study, an acidic heteropolysaccharide fraction, LFP-a1, was purified from Lycii fructus. The preliminary structure of LFP-a1 was characterized. Together with the results from *in vitro* and *in vivo* analyses, the extent of oxidative stress determines, at least in part, the cell fate leading to the activation of mitochondrial-mediated apoptotic pathways in both H_2_O_2_-induced NAFLD model of L02 cells and TAA-induced NAFLD model of zebrafish larvae. Exogenous LFP-a1 successfully protected against oxidative stress-triggered apoptosis, indicating that inhibition of abnormal apoptosis may encode the underlying mechanism contributing to the cytoprotective effect of LFP-a1 during oxidative stress. Clearly, to confirm the protective effect and potential therapeutic significance of LFP-1, there is a need for longer term trails with increased number of larvae. The systematic evaluation of the cluster of oxidative stress-associated, apoptosis-associated, and lipid metabolism-associated biomarkers is further needed to elucidate the incompletely understood molecular mechanisms. Despite the interpretative constraints, our findings underscore the potential of LFP-a1 in preventing or treatment of NAFLD or other oxidative stress-related diseases, which has important implications when proceeding to the next stages of therapeutic approaches.

## Figures and Tables

**Table 1:** FT-IR analysis of functional groups in LFP-a1.

Peak (cm^−1^)	Intensity	Characterization	Functional group
3423	S	-O-H stretching vibration	O-H
2933	W	-C-H stretching vibration	-CH_2_-
1628	S	-C=O stretching vibration	-COOH
1410	M	-C-O stretching vibration	-COOH
1074	S	-C-O-C and C-O-H overlaping vibrations	/

## Data Availability

Data in this study are available from the corresponding author upon request.

## Supplementary Materials

Supplementary Materials Table S1: nucleotide sequences of the primers used to quantify mRNA levels by qPCR.
