# Supplementary material for: Hepatoprotection of Lycii Fructus Polysaccharide against Oxidative Stress in Hepatocytes and Larval Zebrafish
Source: Oxid Med Cell Longev. 2021 Feb 18;2021:3923625. doi: 10.1155/2021/3923625 (PMC7906805; doi:10.1155/2021/3923625)
Supplement: Supplementary Materials — Table S1: nucleotide sequences of the primers used to quantify mRNA levels by qPCR. [file 3923625.f1.docx]

Supplementary Description:

T_ABLE_ S1: Nucleotide sequences of the primers used to quantify mRNA levels by qPCR
